# Trends in epidemiology of Hepatitis B and C Infections in Ilam Province: National Notifiable Diseases Surveillance System data

**DOI:** 10.22088/cjim.9.1.16

**Published:** 2018

**Authors:** Salman Khazaei, Manoochehr Karami, Erfan Ayubi, Abolfazl Mohammadbeigi, Azim Hasanbeigi, Kamyar Mansori, Mohammad Reza Mir-Shekar

**Affiliations:** 1 Department of Epidemiology, School of Public Health, Hamadan University of Medical Sciences, Hamadan, Iran; 2 Department of Epidemiology and Biostatistics, School of Public Health, Tehran University of Medical Sciences, Tehran, Iran; 3 Modeling of Noncommunicable Diseases Research Center, Hamadan University of Medical Sciences, Hamadan, Iran; 4 Department of Community Medicine, Zahedan University of Medical Sciences, Zahedan, Iran; 5 Gastroenterology & Hepatology Diseases Research Center, Qom University of Medical Sciences, Qom, Iran; 6 Deputy of Health, Ilam University of Medical Sciences, Ilam, Iran.; 7 Department of Public Health, School of Public Health, Dezful University of Medical Sciences, Dezful, Iran

**Keywords:** Hepatitis B, Hepatitis C, Incidence, Trend, Iran

## Abstract

**Background:**

Hepatitis B virus (HBV) and hepatitis C virus (HCV) infections are the main causes of severe liver failure worldwide. This study was conducted to evaluate the incidence rate and trend of these infections over six successive years from 2008 to 2013 in Ilam Province, western Iran.

**Methods:**

We studied the registered data of HBV and HCV based on the National Notifiable Diseases Surveillance System in Ilam Province from 2008 to 2013. Incidence rate per 100,000 populations was estimated for HBV and HCV infections through location, years and age groups of patients.

**Results:**

The overall incidence rate per 100,000 populations from 2008 to 2013 for HBV infection was 9.57, 5.83, 16.26, 12.44, 21.89 and 13.93, respectively. The corresponding values for HCV infection were 0.55, 0.72, 1.44, 2.69, 1.24 and 1.93, respectively and these trends for both the HBV and HCV infections were increasing. The major distribution of HBV and HCV infections was 25-44 years of age. Both HBV and HCV infections were more common in males, urban areas and married patients. Forty-one percent of cases were carriers and history of surgery was the common risk factor.

**Conclusions:**

Our results showed that HBV and HCV are prevalent in the middle-age group. Despite effective vaccination against hepatitis B, optimized blood donor screening and better sterilization procedures for blood products, trend of HBV and HCV in Ilam are increasing. Further studies should address the role other risk factors in the trend of HBV and HCV.

Hepatitis B virus (HBV) and hepatitis C virus (HCV) are the main causes of severe liver failure, including hepatocellular carcinoma (HCC) and cirrhosis (1). Approximately 80% of HCC cases are attributed to chronic HBV or HCV infections (2). It is estimated that nearly 350 million people worldwide have chronic HBV. This value for chronic HCV is 170 million (3). HBV is highly contagious and transmitted mainly via blood transfusion, unsafe injection practices, sexual contact and mother to child transmission (4). HCV transition way is similar to HBV and it is estimated that about 3% of the populations in the world are carriers of HCV, with 3 to 4 million new infections per year (5). Unlike HCV, HBV vaccine has been available since 1982, and has a high effect in the prevention of HBV transmission (4). Due to asymptomatic and latent nature of these diseases prior to clinical stage, calculating prevalence rates across the world is difficult (6). The prevalence of HBV infection is very diverse all over the world ranging from highly endemic areas (> 8% infection rate) in South-East Asia and the sub-Saharan regions, intermediate (2-8%) in Mediterranean countries and Japan to low endemic areas (< 2%) in the United States and Western Europe (7). 

WHO estimations show that the Eastern Mediterranean region of WHO has the largest reservoir of HCV globally with the prevalence rate of 11% to 14% (3). The result of the systematic review study showed that in Iran, the prevalence of the HBV infection was estimated 2.14% and ranged from 1.3% in Kermanshah, East Azarbaijan and Isfahan to 6.3% in Golestan province (8), In another study, history of surgery and imprisonment were the main risk factors for the HBV infection (9). The prevalence of HBV infection in general population in Iran is less than 1%, lower than in most of the regional countries (10).

The evaluation of the prevalence and distribution of HBV and HCV is important for the planning of prevention programs and the provision of necessary medical equipment particularly in the case of HBV for the development of vaccination programs (11). Considering the national vaccination programs for hepatitis B, optimized blood donor screening and better sterilization procedures for blood products in Iran, we expect to have a decreasing trend in the incidence rate of diseases in Ilam. Accordingly, this study aimed to address the epidemiological profile of HBV and HCV in Ilam province from 2008 to 2013. 

## Methods

We studied the registered data of the HBV and HCV reported cases to the provincial level of National Notifiable Diseases Surveillance System in Ilam Province from 2008 to 2013. Ilam is located in the west of the country bordering Iraq. The population of the province is approximately 600,000 people (2015 estimate). According to National guideline hepatitis management, notification of HBV and HCV infections in Iran is mandatory (12). Therefore, all public and private laboratories, blood transfusion organization, hospitals and health centers should report all positive test results of serologic markers of HBV and HCV infections to the affiliated district health center monthly. All patients with positive serologic markers for HBsAg or anti-HCV were considered as suspected positive cases of HBV or HCV infections, respectively and were included in the study. To reach the homogenous and generalizable data, those patients who lived in other provinces were excluded from the study. Moreover, to consider only new cases, cases with previous history of disease were excluded and only incident cases in this period enrolled in the study. Then, complete examination forms for identifying detected cases was done by the health staff. This form includes data on demographic characteristics of the patients (age, gender, marital status, and residence), history of high risk behavior, cause of examination and disease situation in other family members with similar situation.

Incidence rate per 100,000 populations estimated for HBV/ HCV infections was disaggregated by location and age group of patients. The average annual rate of reduction/increase (AARR/I) was calculated using a regression analysis to quantify the rate of change of the incidence rate from 2008 to 2013. To this end, the incidence rate of HBV and HCV infection and the year were considered as the dependent and independent variables, respectively. Then, using the coefficient (B) obtained the AARR was calculated with the following formula (13): AARR= 1-EXP(B). Sign of coefficient (B) was accounted for direction of trend. In fact, change in incidence is assumed to take an exponential function. For any given year t, if the incidence is known to be Yt, and the annual rate of reduction or increase is constantly b%, then the incidence of the next year, denoted as Yt+1, can be calculated as: Yt+1 = Yt*(1-b%)

Chi square test was used to compare some infection types (HBV, HCV, HBV & HCV) according to the mentioned variables. Statistical analysis was performed using the Microsoft Excel program and the Stata software, Version 12 (Stata Corp, College Station, TX, USA).

## Results

During the study period, number of 568 patients (514 subjects with HBV infection, 48 subjects with HCV infection and 6 cases of HBV & HCV co-infection) were reported to the provincial level of National Notifiable Diseases Surveillance System in Ilam. Their mean age was 39.12±15.18 years (range 1–87 years).

**Figure 1:**
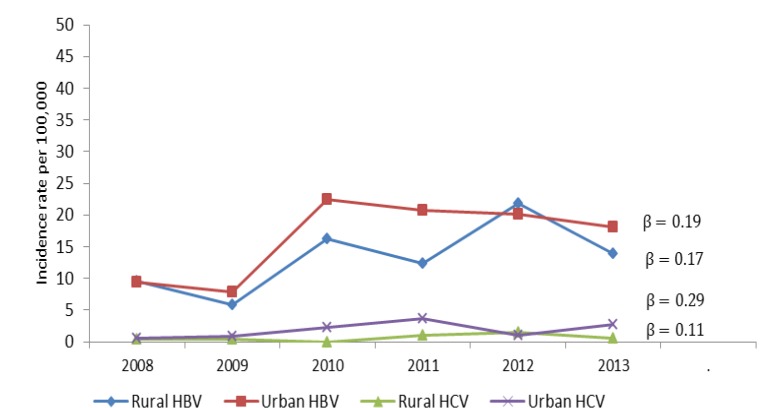
The incidence rate of hepatitis B and hepatitis C virus by location and year

The overall incidence rate pre 100,000 populations from 2008 to 2013 for HBV infection was 9.57, 5.83, 16.26, 12.44, 21.89 and 13.93, respectively. The corresponding values for HCV infection were 0.55, 0.72, 1.44, 2.69, 1.24 and 1.93, respectively. The incidence rates of HBV and HCV according to their location and years of study are shown in figure 1. Finding shows that the overall incidence rate for both HBV (Urban areas: β=0.19, *P*=0.11, rural areas: β=0.17, *P*=0.11) and HCV (urban areas: β= 0.29, *P*=0.11, rural areas: β=0.11, *P*=0.15) is increasing. 

**Figure 2:**
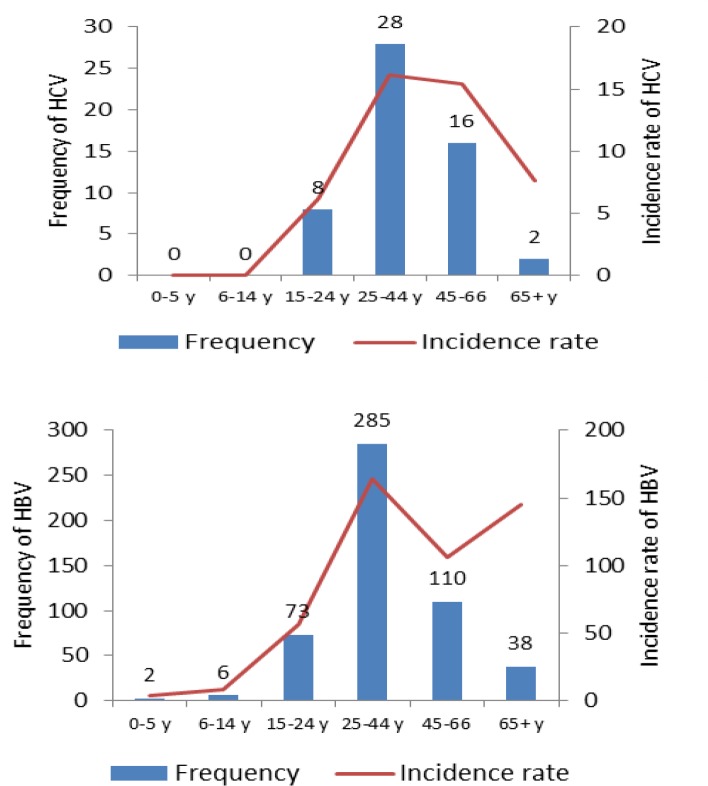
The frequency and incidence rate of hepatitis B and C infection by age group

The absolute frequency distribution of HBV and HCV infections by age and their relevant incidence rates are shown in figure 2. Majority of HBV and HCV infections were observed among individuals who have 25-44 years old. Also, the line curves indicate that the incidence rates of both hepatitis B and hepatitis C are the highest in this age group. High incidence rates of hepatitis B above 65 years old subjects are remarkable.

**Figure 3:**
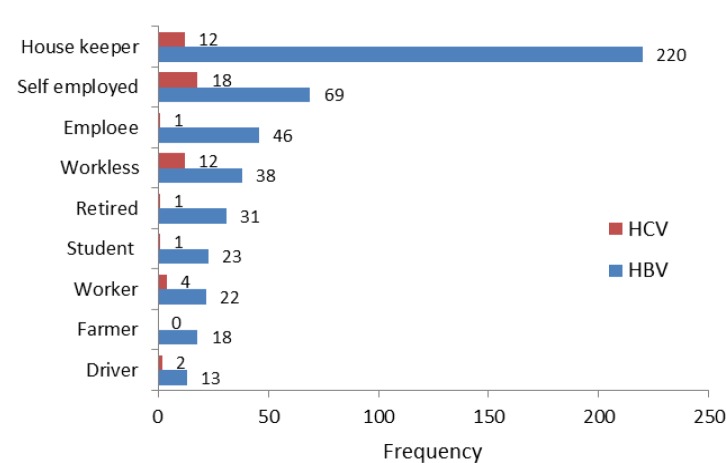
Occupational status of hepatitis B and C infected patients

Figure 3 shows the occupational status of subjects under study. Both hepatitis B and C are more common among women housekeepers and self-employed subjects.

Demographic characteristics of hepatitis B, C and HBV and HCV co-infected patients are shown in Table 1. About 52% (299) cases of the study participants were males, 464 (81.69%) of them were married and 397 (69.89%) lived in urban areas. In 20.77, 17.61, 15.49 and 11.09 percent of subjects, the causes of examination were history pregnancy, volunteer, clinical symptoms and history of high risk behavior. 41.02% of cases were carriers and 27.29% of them were in chronic stage. There was a significant association between infection type and gender, marital status and cause of examination and (p<0.05). Of the 568 cases reported, 274 subjects mentioned history of risk factor related to HBV and HCV. Nonparticipants were similar to participants with respect to age, sex and location. Among subjects who mentioned history of risk factor related to these diseases, surgery was a commonly recognized risk factor for infection identified 18.7% of participants. Infection in other family members (16.3%) and blood transfusion (7.57%) were the other predominant risk factors for infection.

**Figure 4:**
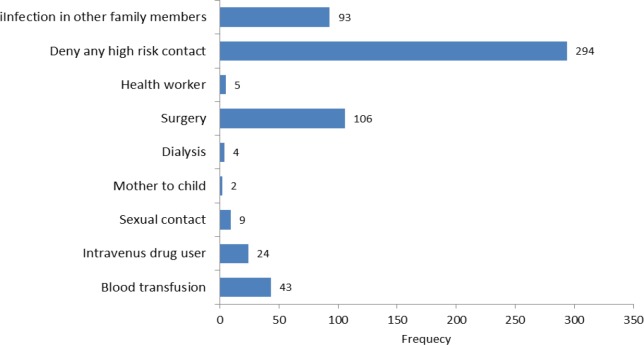
History of risk factors of HBV and HCV infections among patients under study

**Table 1:** Baseline characteristics of hepatitis B, C and HBV and HCV co-infected patients

**Variable**		**Total** **(N=568)**	**HBV** **(N=514)**	**HCV** **(N=48)**	**pvalue** *	**HBV & HCV** **(N=6)**
Gender	Male	299 (52.64)	259 (86.62)	37 (12.37)	<0.001	3 (1.00)
	Female	269 (47.36)	255 (94.80)	11 (4.09)		3 (1.12)
Marital status	Married	464 (81.69)	430 (92.67)	31 (6.68)	0.003	3 (4.90)
	Single	93 (16.37)	75 (80.65)	16 (7.90)		2 (2.15)
	Divorce	11 (1.94)	9 (81.82)	1 (9.09)		1(9.09)
Location	Rural	171 (30.11)	162 (94.74)	8 (4.68)	0.032	1 (0.58)
	Urban	397 (69.89)	352 (88.66)	40 (10.08)		5 (1.26)
Cause of examination	Clinical symptoms	88 (15.49)	85 (96.59)	2 (2.27)	<0.001	1 (1.14)
	High risk behavior	63 (11.09)	41 (65.08)	19 (30.16)		3 (4.76)
	Pregnancy	118 (20.77)	117 (99.15)	0 (0.00)		1 (0.085)
	Volunteer	100 (17.61)	90 (90.00)	9 (9.00)		1 (1.00)
	Other	199 (35.04)	181 (90.95)	18 (9.05)		0 (0.00)

* P values were obtained from HBV and HCV patient comparison based on their baseline characteristics.

## Discussion

HBV is considered as a major public health problem in many countries affecting a large number of populations worldwide. Iran is located in the Middle East, known as an area with an intermediate prevalence of HBV infection (14). In Iran, the mass vaccination program for HBV started in 1993 (15). In the present study, both the information of the 568 HBV/HCV patients in Ilam province for six year surveillance (2008 to 2013) was used. This study indicated that the overall incidence rate of HBV and HCV infection is increasing and the major distribution of HBV and HCV is in 25-44 years group. 90.42% of subjects were HBsAg positive and these infections were more common among males, married patients and those in urban areas. 41.02% of cases were carriers and history of surgery was the common risk factor among the subjects.

Unlike the results of this study, findings of the similar study in Hamedan showed a downward trend of HBV and unsteady trend of HCV infection (16). One reason of this contradiction may be due to the differences in the years of two studies. In return, the results of the another study in Iran was consistent with our results (17). Increasing trend of HCV infection can justify an increase in the number of intravenous drug users (IDUs). In fact, HCV infection is an emerging disease in our country due to IDUs (10). In Latin America in most parts the prevalence has remained in a flat period or increasing, as injection drug use is not common in these countries, therefore other risk factors play a major role in new infections (18). In some studies, increase in the total number of donations was associated with the increasing incidence of infection in those years (19). The increase in blood donation, cesarean delivery and surgery in a year can be associated with increased incidence. It is necessary to note that this increase in trends was not statistically significant. In this study, the major distribution of HBV and HCV was in 25-44 years group. The low incidence rate of HBV infection in less than 25 years of age is attributed to mass vaccination against HBV infection began in 1993 among neonates and immunity as well (14). The low incidence rate of HCV in this age group could be due to the usually initiation of drug injection that occurred among injection drug users (20, 21). Although few subjects have mentioned the history of drug use as a risk factor, it should be noted that in this study about 57% of them refused to answer this question. Given that injecting drug use is an illegal and stigma impacted behavior, therefore most of them were reluctant to answer this question, likewise the sex workers (22). Health planning should note the high incidence rate of HBV and HCV infections in the age group over 65 years in spite of its low frequency.

More than 16% of participants had positive HBV or HCV case in their household. Some studies have shown a relatively higher prevalence in the household members with the HBV or HCV positive patients (14, 23). One reason may be due to the fact that they exist in the same community with the same risk factors as their patient in the family. Another reason may be due to pharmaceutical injection for patients at home or exposure to the patient’s contaminated needles or syringes. In terms of transmission via blood transfusion due to precise would be donor screening process and the elimination of high-risk donors after 1996 in Iran, this way of transmission would be negligible (24). Fortunately, immunization of high-risk groups, such as health care workers, other family members of positive case and blood product recipients against HBV infection, is in process according to the recommendation of national vaccination program (15). There was no tremendous difference in the incidence of the disease between males and females. This might be related to the fact that cesarean surgery belongs to women, furthermore, because of the tests done during pregnancy, women attend more screening than men. In return, needle sharing among intravenous drug abusers and imprisonment are more common in males (21). The result of the population-based study in three provinces in Iran showed that HCV is more common among males but for HBV, there was no significant preponderance regarding sex (17). Some studies indicated higher prevalence of HBsAg among males (25, 26). Regarding residency, in this study, HBV / HCV infection was higher in urban areas. One reason for this difference is due to the rural and urban population composition difference in Ilam province (urban/ rural ratio: 1.84). In addition, HBV and HCV infections are more common in married participants. One reason is that, most married people have not been included in the national vaccination programs and are not protected against the infection; another cause is due to sexual contact between couples. This result is consistent with the findings of other studies in the east of Iran (27). 

The first limitation of this study concerns the collected data based on passive surveillance system, therefore underreporting and lack of completeness are inevitable in this study (28). The second limitation is that, HBV and HCV infections usually affect chronic patients without symptoms and patients usually accidentally diagnosed during blood donation, during pregnancy screening or due to a positive case in other family members, hence the results strongly suggest underestimation (29, 30). Third, confirmatory tests have not been used for definitive diagnosis of subjects including recheck of HBsAg for chronic HBV infection diagnosis and more specific tests to confirm HCV infection, thus some reported cases may be false positive. And fourth is owing to self-reported answer to some questions, consequently, some results are prone to information bias. Despite the limitations of passive surveillance systems like low sensitivity, alongside the low cost and easy to carry out, these systems are useful in monitoring trends over time and providing critical information to monitor community health (31), which is consistent with the objectives of our study.

In conclusion, our results showed that HBV/HCV infections are concentrated in the middle age group. Despite vaccine effectiveness against hepatitis B, optimized blood donor screening and better sterilization procedures for trends in blood products of HBV and HCV infections in this study are increasing, as a result, the other risk factors may play a major role in new infections, So, there is a need to do more studies in this regard. Immunization against HBV infection for high-risk groups, such as health care workers, other family members of positive cases and blood product recipients who have no history of vaccination, should be done more seriously.
